# Supplementary material for: Epigenetic Biomarkers as Diagnostic Tools for Neurodegenerative Disorders
Source: Int J Mol Sci. 2021 Dec 21;23(1):13. doi: 10.3390/ijms23010013 (PMC8745005; doi:10.3390/ijms23010013)
Supplement: Supplementary file 1 [file ijms-23-00013-s001.zip › ijms-1492620-supplementary.pdf]

SUPPLEMENTARY

EPIGENETIC BIOMARKERS AS DIAGNOSTIC TOOLS FOR NEURODEGENERATIVE DISORDERS

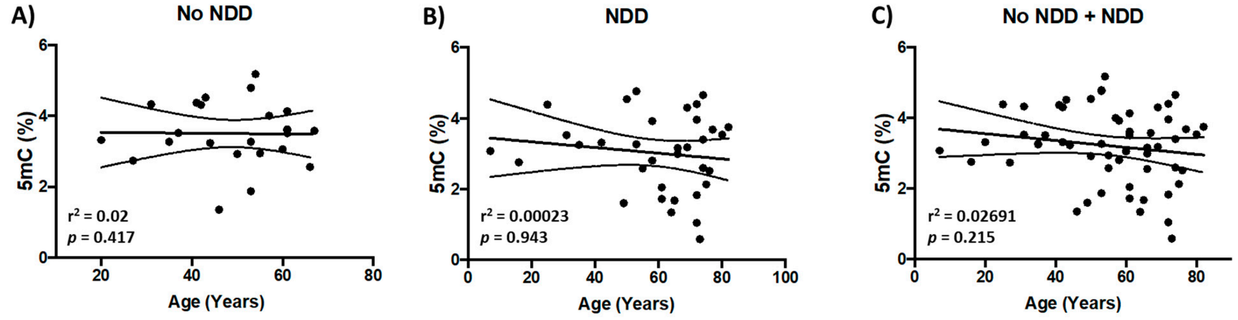

Figure S1. Correlation analysis to test the association between 5mC levels and age in blood samples from patients with and without NDDs. Global DNA methylation (5mC, %) levels were measured colorimetrically in buffy coat samples from (**A**) healthy individuals (n = 25; p = 0,943; r^2 = 0,00024), (**B**) patients with NDDs (n = 35; p = 0.417; r^2 = 0.02) and (**C**) no-NDD+NDD patients (n = 60; p =0.215; r^2 = 0.02691). Data points represent values of individual patients; unpaired t tests were used to determine significance between groups. Solid line, linear regression; stippled lines, 95% confidence interval bands. 5mC, 5-methylcytosine; NDD, patients with neurodegenerative diseases; no-NDD, individuals with no NDDs.

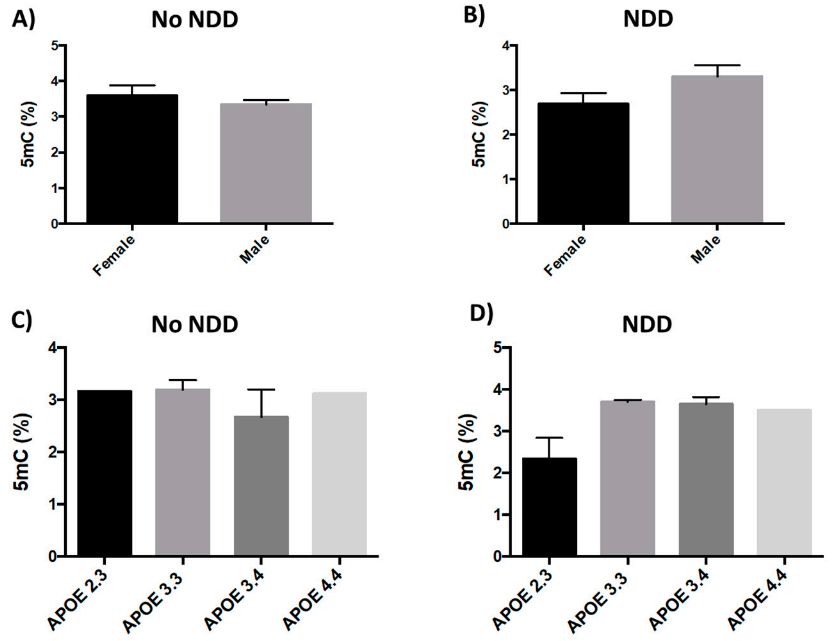

Figure S2. Analysis of global DNA methylation across gender and *APOE* genophenotypes in blood samples from healthy subjects and patients with NDDs. (**A**) Global DNA methylation (5mC, %) levels were measured colorimetrically using buffy coat samples from healthy (No-NDD) individuals (n = 10 males; n = 15 females), and (**B**) patients with NDDs (n = 18 males; n = 16 females). 5mC levels were determined for patients with the *APOE* 2.3, *APOE* 3.3, *APOE* 3.4 and *APOE* 4.4 genophenotypes in (**C**) healthy and (**D**) patients with NDDs. For *APOE* 2.3, n = 2 no NDD, n = 1 NDD; *APOE* 3.3, n = 4 no NDD, n = 7 NDD; *APOE* 3.4, n = 3 no NDD, n = 6 NDD; For *APOE* 4.4, n = 1 no NDD, n = 1 NDD. Data are presented as the mean ± S.E.M; unpaired t tests. 5mC, 5-methylcytosine; APOE, apolipoprotein E; NDD, patients with neurodegenerative diseases; no-NDD, individuals with no NDDs.
